# Nanofiber-expanded human CD34^+^ cells heal cutaneous wounds in streptozotocin-induced diabetic mice

**DOI:** 10.1038/s41598-019-44932-7

**Published:** 2019-06-10

**Authors:** Suman Kanji, Manjusri Das, Matthew Joseph, Reeva Aggarwal, Sudarshana M. Sharma, Michael Ostrowski, Vincent J. Pompili, Hai-Quan Mao, Hiranmoy Das

**Affiliations:** 1 Department of Internal Medicine, Wexner Medical Center at The Ohio State University, Columbus, Ohio USA; 2 Hollings Cancer Center, Medical University of South Carolina, Charleston, SC USA; 3 Institute for NanoBioTechnology, Department of Materials Science and Engineering, Johns Hopkins University, Whiting School of Engineering, Baltimore, MD USA; 4 Department of Pharmaceutical Sciences, School of Pharmacy, Texas Tech University Health Sciences Center, Amarillo, Texas USA

**Keywords:** Stem cells, Haematopoietic stem cells

## Abstract

Despite advances in diabetic wound care, the significant number of amputations that occur every year demands more effective therapeutics. Herein, we offer an aminated polyethersulfone nanofiber-expanded human umbilical cord blood-derived CD34^+^ cells (henceforth CD34^+^ cells) effective therapy, tested in cutaneous wounds developed in streptozotocin-induced diabetic NOD/SCID mice. We show that systemic administration of CD34^+^ cells homed to the wound site and significantly accelerated wound closure. Wound closure was associated with improved re-epithelialization and increased neovascularization; and with decreased sustained pro-inflammatory activity of NF-κB and its downstream effector molecules TNF-α, IL-1β, and IL-6 at the wound bed. This finding was further supported by the observation of a decreased number of myeloperoxidase positive neutrophils, and concomitantly increased levels of IL-10. In addition, improved granulation tissue formation was observed along with higher collagen deposition and myofibroblasts and decreased expressions of MMP-1. Mechanistically, CD34^+^ cells reduced the level of MMP-1 expression by inhibiting recruitment of NF-κB to the MMP-1 promoter site in dermal fibroblasts. In summary, we provide evidence of a novel nanofiber-expanded CD34^+^ stem cell therapeutic development for treating diabetic wounds by defining their cellular and molecular mechanisms.

## Introduction

Diabetic foot ulcers are a growing issue due to worldwide severe increase in diabetes and obesity incidences^1,2^. They affect over 20.8 million people in the USA, and over 170 million worldwide, and these numbers are projected to be doubled by 2030^3^. Diabetes associated chronic wounds are projected to occur in 15% of all patients with diabetes followed by lower limb amputations in 84% of these patients^3^. Diabetes-mediated chronic wounds are a leading cause of hospitalization and morbidity, and cost $25 billion per annum in the US alone^1^.

Sustained inflammation is a major cause of delayed wound healing in diabetes. Hyperglycemia-induced reactive oxygen species activate innate immune responses for sustained hyperactivation of NF-κB, which mediates inflammation^4,5^. In addition, activated neutrophils also contribute to persistent inflammation^6^. Angiogenesis regulates tissue regeneration in wound healing, and endothelial cells are primarily involved in this process; however, hyperglycemia dysregulates normal angiogenesis^7^. In addition, granulation tissue organization, in which dermal fibroblasts regulate wound-healing process by secreting, contracting, and remodeling extracellular matrices (ECMs) are also impaired by diabetes. Hyperglycemia affects normal proliferative, synthetic, and secretory functions of fibroblasts, and disrupts the secretion of ECMs^8,9^. The number of fibroblasts is critically reduced in wounds of diabetic individuals^10^. High concentration of TNF-α is one of the causes of delayed healing in diabetic wounds^11^. A poor ECM formation in diabetic wounds is not only due to inadequate synthesis of ECM proteins but also to an increased rate of ECM degradation by proteolytic enzymes like matrix metalloproteinases (MMPs); MMP1 is one of the predominant collagenases found in chronic wounds^12^.

Current advanced diabetic wound care and therapy provide only partial relief and do not cure the disease, as a result, diabetic wounds either do not heal or heal very slowly^1^. Efforts have been made to develop more effective therapeutics to cure wounds through tissue regeneration. We previously demonstrated that systemic administration of nanofiber-expanded CD34^+^ cells accelerated acute wound closure in NOD/SCID mouse model^13–15^. However, the effect of nanofiber-expanded CD34^+^ hematopoietic stem cell therapy in diabetic cutaneous wound is yet to be defined. The current study was undertaken to investigate the efficacy of nanofiber-expanded CD34^+^ stem cells in mediating healing of wound in a comorbid diabetic condition. This study also focuses on revealing cellular and molecular mechanisms involved in CD34^+^ cell therapeutic effects. In addition, efforts were given to delineate epigenetic regulation of factors responsible in maintaining non-healing conditions in diabetic wounds.

## Materials and Methods

All methods were carried out in accordance with relevant guidelines and regulations. The Ohio State University Medical Center institutional review board (IRB) and institutional animal care and use committee (IACUC) approved all experimental protocols. Informed consents were obtained from all human subjects.

### CD133^+^ cell isolation and expansion

Human umbilical cord blood was collected freshly from The Ohio State University Medical Center with an approved IRB after receiving written informed consent from donors, and all research was performed in accordance with the University guidelines. Collected blood was processed following protocol described earlier^16^. In brief, mononuclear cells were collected by Ficoll separation, followed by CD133^+^ cells isolation using AutoMACS device (Miltenyi Biotec, Auburn, CA), and purity of the isolated cells and phenotypes after expansion, were determined by flowcytometry as described later. Isolated CD133^+^ cells were expanded according to the protocol described previously^16^. Briefly, eight hundred CD133^+^ cells were seeded onto each well of a 24-well cell culture plate containing glued aminated polyethersulfone (PES) nanofiber scaffold (produced in Hai-Quan Mao’s, lab) in 600 μl of StemSpan SFEM, serum-free expansion medium (Stem Cell Technologies, Vancouver, BC, Canada), with essential supplements. Cells were cultured for 10 days at 37 °C in 5% CO_2_ without changing medium. Cell phenotypes were confirmed by flowcytometry before conducting experiments.

### Flowcytometry

PES coated nanofiber expanded cells were incubated at 4 °C for 30 min in 2% FBS Hanks’ buffer in the presence of various antibody combinations as described earlier^16,17^. Anti-CD34-FITC, and anti-CD133-PE were purchased from Miltenyi Biotec Inc. Fluorescently labeled (PE/FITC) antibodies for other cell surface markers (CXCR4, von Willebrand Factor (vWF), CD31, CD14, CD11b, MHC class I, MHC class II, CD69, CD3, and CD86) were purchased from BD Biosciences (USA). Cells were analyzed by two-color flow cytometry on a FACS Calibur analyzer (BD Biosciences). Relevant isotype controls were used to confirm the specificity and compensation setting. Data analysis was performed with BD Cell Quest software.

### GFP labeling of nanofiber expanded CD34^+^ cells

Using a human CD34 cell Nucleofector kit (Amaxa Inc.) nanofiber-expanded CD34^+^ cells were transfected with GFP containing vector (pmaxGFP) following the manufacturer’s protocol^16^. Cells were cultured in serum-free complete expansion media for overnight after transfection.

### Generation of diabetes in NOD/SCID mice

All murine experiments were performed complying with the protocols approved by the Institutional Animal Care and Use Committee of The Ohio State University. Immunocompromised NOD/SCID mice were purchased from Jackson laboratory (Bar Harbor, ME) and 8-10-week-old male mice were used in this study to avoid any hormonal influences during the healing process. After a week of acclimatization, mice were fasted for 4 hours before injecting streptozotocin (STZ) as described earlier^18^. Briefly, 50 mg/kg doses of STZ (Teva parenterals Inc, Irvine, CA) were dissolved in citrate buffer (pH 4.2) and injected intraperitoneally for 5 consecutive days. To assess development of diabetes, non-fasting blood glucose levels were monitored every week by using AlphaTRAK blood glucose monitoring system (Abbott Laboratories, North Chicago, IL, USA). A drop of blood was collected from the tip of the tail after brief anesthesia and placed on the blood glucose monitoring system. The resulting blood glucose level was above 300 mg/dl, and was considered a diabetic phenotype in NOD/SCID mice.

### Generation of full thickness excisional cutaneous wound and transplantation of CD34^+^ cells

After four weeks of STZ injection, cutaneous wounds were developed in diabetic NOD/SCID mice that contain blood glucose levels above 300 mg/dl. A full-thickness 8-mm punch biopsy (Acuderm Inc. Fort Lauderdale, FL) was made in each mouse on the dorsal skin under anesthesia after shaving and wiping with Betadine solution. Nanofiber-expanded CD34^+^ cells (0.5 × 10^6^ cells/mouse) were injected into each group of mice through the lateral tail vein with serum free media of 200-μl volume after 2 h of cutaneous excision. Control mice received vehicle only. Mice were sacrificed on 5th, 10th and 15th days after wounding. Skin samples including the wound and 2 mm of the surrounding skin were harvested at each time point.

### Evaluation of wound area

Wound healing process was imaged with a digital camera (Sony cyber-shot DSC-H10) from a fixed distance on every alternate day until day 15 of post-wounding keeping day 0 images as control. The rate of wound closure was measured by tracing the wound area onto an acetate paper following earlier studies^13,14^. The tracings were digitalized after scanning, and the areas were measured by using the UTHSCA (University of Texas Health Science Center at San Antonio) image tool (Version 3.00), and shown as percent wound closure. Data collection and wound measurements were performed in a blinded fashion. Following formula was used to calculated percentage of wound closure: (Area of wound on day 0 - Area of wound on day of examination)/Area of wound on day 0 × 100).

### Immunohistochemistry

Skin tissues were harvested after sacrifice of mice, and part of the tissue was fixed in 10% formalin-PBS buffer before processing. Tissues were then embedded in a paraffin block and were sectioned with 4 µm thicknesses. After de-paraffinization, sections were stained with hematoxylin & eosin (H&E) or Masson’s trichrome following standard methods. Before immunostaining, antigen was retrieved by baking with a microwave for 5 min in citrate buffer (pH 6.0). After non-specific blocking, staining was performed using VECTASTAIN Elite ABC kits following manufacturer’s protocol (Vector laboratories Inc, Burlingame, CA) with von-Willebrand Factor (vWF) (Dako, Carpentaria, CA) primary Ab or myeloperoxidase (MPO) primary Ab, and detected with 3,3′-Diaminobenzidine (DAB). Visualization was performed under a microscope (Axioplan2; Carl Zeiss) and images were captured with Zeiss, Axiovision imaging software (Carl Zeiss). Data was analyzed by an image analysis software program (ImageJ, NIH) following color deconvolution method^13^.

### Quantitative RT-PCR analysis

Total RNA extraction was performed from wound-edge tissues that were harvested on days 5 and 10 using TRIzol RNA extraction method (Invitrogen). The reverse-transcription of one µg of mRNA was performed using the ‘High Capacity cDNA Reverse Transcription Kit’ (Applied Biosystems, Foster City, CA). One 20th of the cDNA was used for the real time PCR analysis. Reactions were conducted in SYBR Green PCR master mix (Applied Biosystems) using a Light Cycler 480 (Roche Applied Science) detection System. The primers were used as follows: Mouse (m) Interleukin-1β (IL-1β), forward 5′-TTGAAGTTGACGGACCCCAA-3′, reverse 5′-TGCTGCTGCGAGATTTGAAG-3′; m-Tumor necrosis factor- α (TNF-α), forward 5′-CAACGGCATGGATCTCAAAGAC-3′, reverse 5′-AGATAGCAAATCGGCTGACGGT-3′; m- Interleukin-10 (IL10), forward 5′-TAGAGCTGCGGACTGCCTTCA, reverse5′-ATGCTCCTTGATTTCTGGGCCAT; m-β- actin, forward 5′-TGTGATGGTGGGAATGGGTCAGAA-3′, reverse 5′-TGTGGTGCCAGATCTTCTCCATGT-3′; mRNA expression levels were normalized with β-actin.

### Western blot analysis

Expression of various proteins in murine wound tissue with or without CD34^+^ cell therapy were analyzed by Western blot (WB) methods following standard procedures. Primary antibodies used were MMP-1, MMP-2, p65 (all from Santa Cruz, CA), β-actin, GAPDH (all from Cell Signaling, Beverly, MA) and αSMA (Sigma, USA). Mouse, rabbit IgG-HRP conjugated (Cell Signaling, Beverly, MA) secondary Abs were used, and specific bands were detected by enzyme-linked chemiluminescence (Pierce, IL) methods. Densitometric analysis of developed bands was performed by a standard scanner and analyzed with UN-SCAN-IT (gel 6.1 version) software. Relative density was calculated by using respective GAPDH/ β-actin levels.

### Total collagen assay

Sircol Collagen Assay kit was used for colorimetrically measuring collagen (Newtown Abbey, UK) following manufacturer’s protocol. In brief, recommended amount of Sircol dye were added to the tissue extracts, mixed for 30 min by stirring at room temperature, and centrifuged at 12,000 rpm for 10 min. Absorbance of the bound dye was measured at 555 nm wavelength using a spectrophotometer. The amount of collagen in each sample was adjusted to the total protein, which was estimated by using the BCA Protein Assay kit (Pierce, Rockford, IL, USA). Collagen concentrations were expressed as μg collagen/mg of total protein.

### Fibroblast cell culture

A primary human dermal fibroblast cell line was established from skin punch biopsies of a healthy donor, who was undergoing for an accidental surgery following the IRB approval from The Ohio State University. Primary human dermal fibroblast cells (generous gift from Dr. Heather M. Powell, The Ohio State University, Columbus, Ohio) were maintained in Dulbecco’s modified Eagle’s medium (DMEM) (Invitrogen Corporation, Carlsbad, CA, USA), supplemented with 4% fetal calf serum (FCS) (Sigma-Aldrich, St. Louis, MO, USA), 2 mM glutamine (Invitrogen Corporation), 5 μg/ml insulin (Sigma-Aldrich), 0.5 μg/ml Hydrocortisone (Sigma-Aldrich), 0.1 mM ascorbic acid-2-phosphate (Sigma-Aldrich), 50 U/ml penicillin, and 50 μg/ml streptomycin (Invitrogen) and grown in 5% CO_2_ at 37 °C and used from passage 3 to 7.

### Co-culture of CD34^+^ cells and dermal fibroblast

Primary human dermal fibroblasts were co-cultured with CD34^+^ cells in a well of a 6-well plate using fibroblast culture media. Dermal fibroblasts were mostly seeded at 3 × 10^5^ cells/well in a 6-well plate and cultured them in DMEM media for 6 hours. Fibroblast cells were serum starved for 24 h prior to any experiments. After serum starvation, part of CD34^+^ cells was stimulated with 10 ng/ml human recombinant (r)TNF-α (PeproTech, Rocky Hill, NJ, USA). The following groups were devised: untreated controls, fibroblast treated with rTNF-α (10 ng/ml), fibroblast treated with rTNF-α (10 ng/ml) with an added CD34^+^ cells, fibroblast co-cultured with CD34^+^ cells only for 30, 60 and 90 min time points.

### RNA analysis for MMP-1 expression

Dermal fibroblasts were seeded at 3 × 10^5^ cells/well in a 6-well plate and cultured in DMEM media for 6 hours. Fibroblast cells were then serum starved for 24 h prior to any experiment. Fibroblasts were then treated with 10 ng/ml rTNF-α in the presence or absence of CD34^+^ cells for 60 and 90 min. Total RNA was extracted and quantitative RT-PCR was performed as described above using an ABI step one plus real-time PCR system (Applied Biosystems). Commercially designed inventoried Taqman gene expression assays and fast start Universal probe master mix (Roche) were used for measuring MMP-1 gene expression. Results were normalized by GAPDH expression level.

### Chromatin immunoprecipitation (ChIP) analysis

ChIP experiments and analysis were performed as described previously^19^. Briefly, primary dermal fibroblasts were plated at a density of 5 × 10^6^ cells in a 10-cm dish and treated with rTNFα for 1 h after overnight starvation of cells. After incubation with CD34^+^ cells, by gentle washing CD34^+^ cells were removed (for treatment group) and fibroblasts were cross-linked with 1% final concentration of formaldehyde at 37 °C for 10 min and stopped by addition of glycine to 125 mM final concentration before harvesting. Soluble chromatin was prepared by sonication using a Branson 250 digital sonifier (Branson Ultrasonics, Danbury, CT) to an average DNA length of 200–1000 bp. One sixth of the sheared soluble chromatin was pre-cleared with tRNA-blocked Protein G-agarose, and 10% of the pre-cleared chromatin was set aside as input control. Immunoprecipitation was carried out with 3 µg of p65 antibody for overnight incubation at 4 °C. Immune complexes were pulled down using Protein G-agarose beads, washed, and eluted twice with 250 µl of elution buffer (0.1 M NaHCO_3_, 1% SDS), and cross-linking was reversed by 200 mM NaCl at 65 °C overnight in presence of 20 µg of RNase A (Sigma). DNA was purified after proteinase K treatment (Invitrogen) with the Qiagen PCR purification kit following manufacturer’s protocol. Samples were analyzed by real-time PCR by the recommended probe for the MMP-1 locus from Roche universal probe library (Roche Diagnostics, Indianapolis, IN) using the Fast start TaqMan master kit (Roche Diagnostics). The threshold for the studied promoter was adjusted by the input value, and represented as relative abundance.

### Statistical analysis

All quantitative data are represented as mean ± SEM unless mentioned. Analysis was performed between different groups using 2-tailed student’s t-test. Statistical significance was set at a value of p < 0.05.

## Results

### Isolation, expansion, characterization, and labeling of human umbilical cord blood-derived progenitor cells

We expanded AutoMACS isolated CD133^+^ cells from freshly collected human umbilical cord blood on aminated polyehersulfone nanofiber scaffold coated coverslips, which are glued on each well of a 24-well plates. After 10 days of culture of 20,000 CD133^+^ cells per plate, we obtained almost a total of 5 million expanded cells (~250-fold amplification). Flowcytometric analysis revealed that around 87% cells were positive for CD34 (Supplemental Fig. 1) after nanofiber-mediated expansion. Around 94% expanded cells expressed promigratory and pro-adhesive molecule such as CXCR4. Moderate expressions of other hematopoietic markers were also observed in nanofiber-expanded cells, such as, CD14, CD86, vWF, CD31, indicating that these expanded cells retain their hematopoietic progenitor characteristics.

### Generation of diabetes in NOD/SCID mice

**Figure 1:**
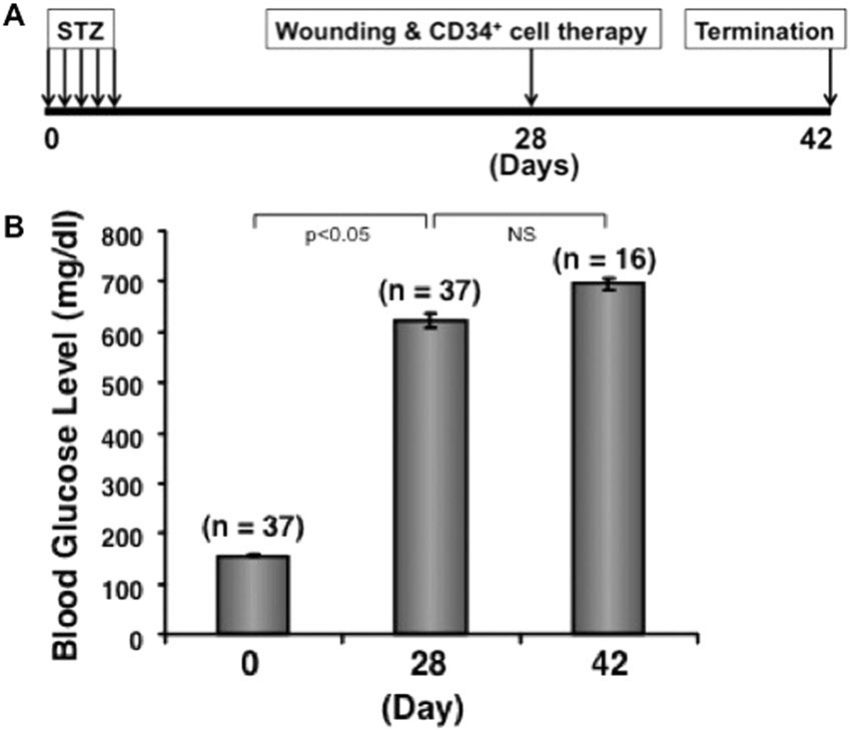
Induction of diabetes in NOD/SCID mice. Diabetes was induced in NOD/SCID mice using STZ, and non-fasting blood glucose levels were measured on day 0 and once a week after cell therapy until the end of the study. Data presented at 3 different time points with ± SEM.

To evaluate the therapeutic efficacy of CD34^+^ cell-mediated wound healing in diabetic NOD/SCID mice, we induced diabetic condition in mice with injection of STZ for 5 days (Fig. 1A). The average non-fasting level of blood glucose was 156.1 mg/dl without STZ injection (Fig. 1B). Upon induction the level reached to 621.8 mg/dl on day 28 before the wound was created in these mice (Fig. 1B). These mice maintained their stable hyperglycemia till the end of the study (day 42), averaging blood glucose level at 695.6 mg/dl (Fig. 1B).

### Effect of CD34^+^ cell therapy in wound closure in diabetic mice

**Figure 2:**
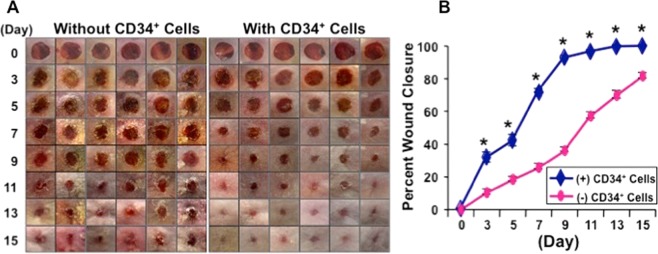
Nanofiber-expanded CD34^+^ cell therapy accelerated wound healing in diabetic NOD/SCID mice. (**A**) Morphological images of cutaneous wounds (8 mm punch biopsies) at various time points during the course of healing in the presence or absence of nanofber-expanded CD34^**+**^ cell therapy. (**B**) Graphical presentation of cumulative measurement of wound area at various time points. Star (*) indicates statistically significant (p < 0.05) improvement in wound closure after cell therapy. Data are presented as mean ± SEM (n = 8).

Morphological image analysis revealed that nanofiber-expanded CD34^+^ cell infusion enhanced wound healing at any given day of the healing process, and that the complete wound closure was observed within 11 days of therapy; whereas without cell therapy most of the wounds were open until 15 days post-wounding (Fig. 2A). Cumulative data analysis showed a statistically significant wound closure at any time points tested after CD34^+^ cell therapy compared to vehicle-treated control (Fig. 2B) indicating the efficacy of CD34^+^ cell therapy for cutaneous wound in the comorbid diabetic condition.

### Effect of CD34^+^ cell therapy in cellularization of cutaneous wound

**Figure 3:**
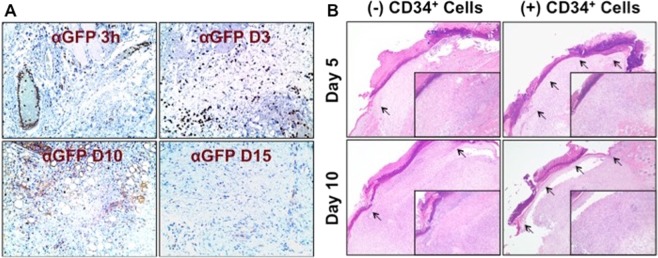
Nanofiber-expanded CD34^+^ cell therapy homed to the wound bed and improved re-epithelialization and granulation tissue formation. (**A**) GFP labeled nanofiber-expanded CD34^+^ cells were detected in wound bed at various time points. (**B**) Hematoxylin and eosin staining of wound sections of the animals with or without CD34^+^ stem cell therapy. Insets show higher magnification of the specific area. Arrowheads indicate epithelialization of the wound tissue.

To determine migration of systemically transplanted nanofiber-expanded CD34^+^ cells in diabetic immunocompromised mice, we found that cells homed to the wound area within 3 h and stayed in the wounded tissue (Fig. 3A). Histological evaluation revealed a greater granulation tissue formation in the wound sections at day 5 or day 10 of animals treated with nanofiber-expanded CD34^+^ cells compared to controls. Complete re-epithelialization was observed in animals treated with CD34^+^ cell at days 5 to 10 as marked by the arrows, whereas initiation of re-epithelialization in the control diabetic wounds was observed at the wound junction on day 5. However, re-epithelialization did not progress in control diabetic wounds till day 10, as re-epithelization was observed only near the wound junction indicated by arrow (Fig. 3B). This result indicates that treatment with CD34^+^ cells improved wound pathology in the diabetic condition.

### Effect of CD34^+^ cell therapy on wound inflammation

**Figure 4:**
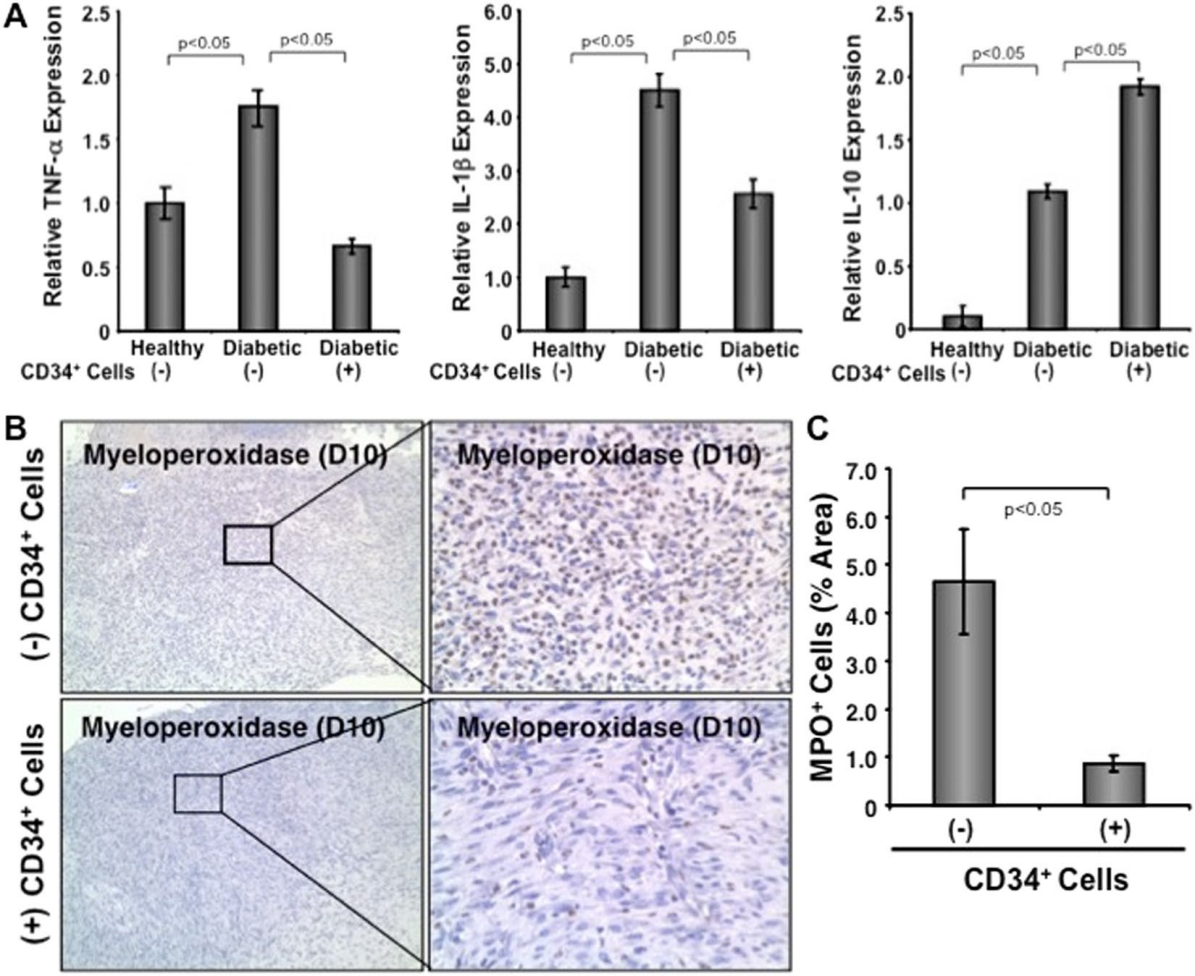
CD34^+^ cell therapy reduced sustained inflammation and neutrophil infiltration within the wounds. (**A**) Graphical presentation of quantitative PCR analysis of stated gene expression in wound tissue with or without CD34^**+**^ cells therapy on day 10. Values were normalized to β-actin level with mean ± SEM (n = 3). (**B**) Formalin fixed paraffin-embedded wound tissues were stained with anti-myeloperoxidase (dark brown) with or without CD34^**+**^ cells therapy on day 10. Right panels are the zooms of the boxed area of images. **C**. Bar graph shows quantitation of neutrophils of 10 randomly chosen high power microscopic fields within the sections. Data are presented as mean ± SEM (n = 3).

To investigate whether CD34^+^ therapy has any role in regulating inflammation in the diabetic wound bed, some of the important proinflammatory and anti-inflammatory genes were verified in the wound tissues after CD34^+^ stem cell therapy. Analysis of quantitative RT-PCR results showed that there was a significant decrease in expression of proinflammatory factors like IL-1β and TNF-α in wound tissues of the animals treated with nanofiber-expanded CD34^+^ cells at day 10 compared to the animals treated with vehicle only (Fig. 4A). Conversely, anti-inflammatory factor IL-10 was increased in wound tissues of the nanofiber-expanded CD34^+^ cells-treated animals compared to the wounds of control animals (Fig. 4A).

In addition, we verified neutrophil infiltration using the specific marker myeloperoxidase by immunohistochemical analysis of wound tissue sections. Immunohistochemical analysis showed a significantly (p < 0.05) reduced number of myeloperoxidase positive neutrophils present at the wound bed at day 10 after CD34^+^ cell treatment compared to controls, indicating that CD34^+^ cells regulate neutrophil infiltration and presence at the diabetic wound tissues (Fig. 4B,C). These results show that CD34^+^ cell therapy reduced proinflammatory activity by decreasing neutrophil infiltration and by reducing proinflammatory cytokine expression in the diabetic wound bed, with concomitantly increased expression of anti-inflammatory IL-10 molecule.

### Effect of CD34^+^ cell therapy on wound vascularization

**Figure 5:**
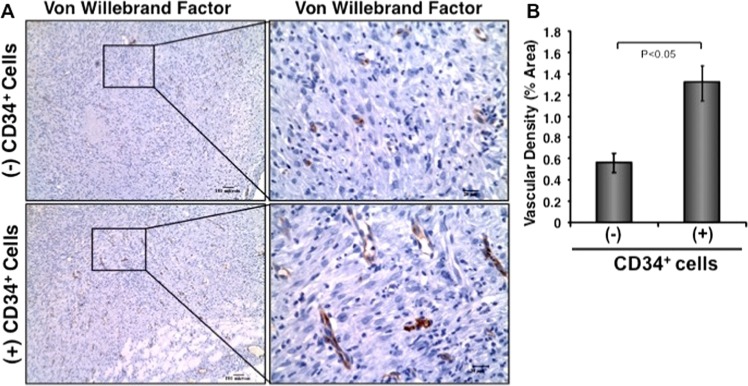
Enhanced neovascularization in diabetic tissues after CD34^+^ cell therapy. (**A**) Formalin fixed paraffin-embedded wound tissues were stained using anti-von Willebrand factor (dark brown) Ab at day 10 of post cell therapy. Right panels are the zooms of the boxed area of images. (**B**) Bar graph shows quantitation of vascular density (in percent area). Data are presented as mean ± SEM (n = 3).

To investigate efficacy of the infused CD34^+^ cells on restoration of wound angiogenesis in diabetic condition, we stained wound sections immunohistochemically using von Willebrand factor, a vascularization marker. Immunohistochemical analysis demonstrated that an increased neovascularization in wounds of diabetic mice after treatment with nanofiber-expanded CD34^+^ cells compared to control mice (Fig. 5A). Quantification of wound vascularization showed a 2.5-fold increase in vascular density in CD34^+^ cell treated wounds compared to control wounds (Fig. 5B), indicating that CD34^+^ cell therapy support wound vascularization in diabetic condition, which further contributed to accelerated healing.

### Effect of CD34^+^ cell therapy on extracellular matrix synthesis

**Figure 6:**
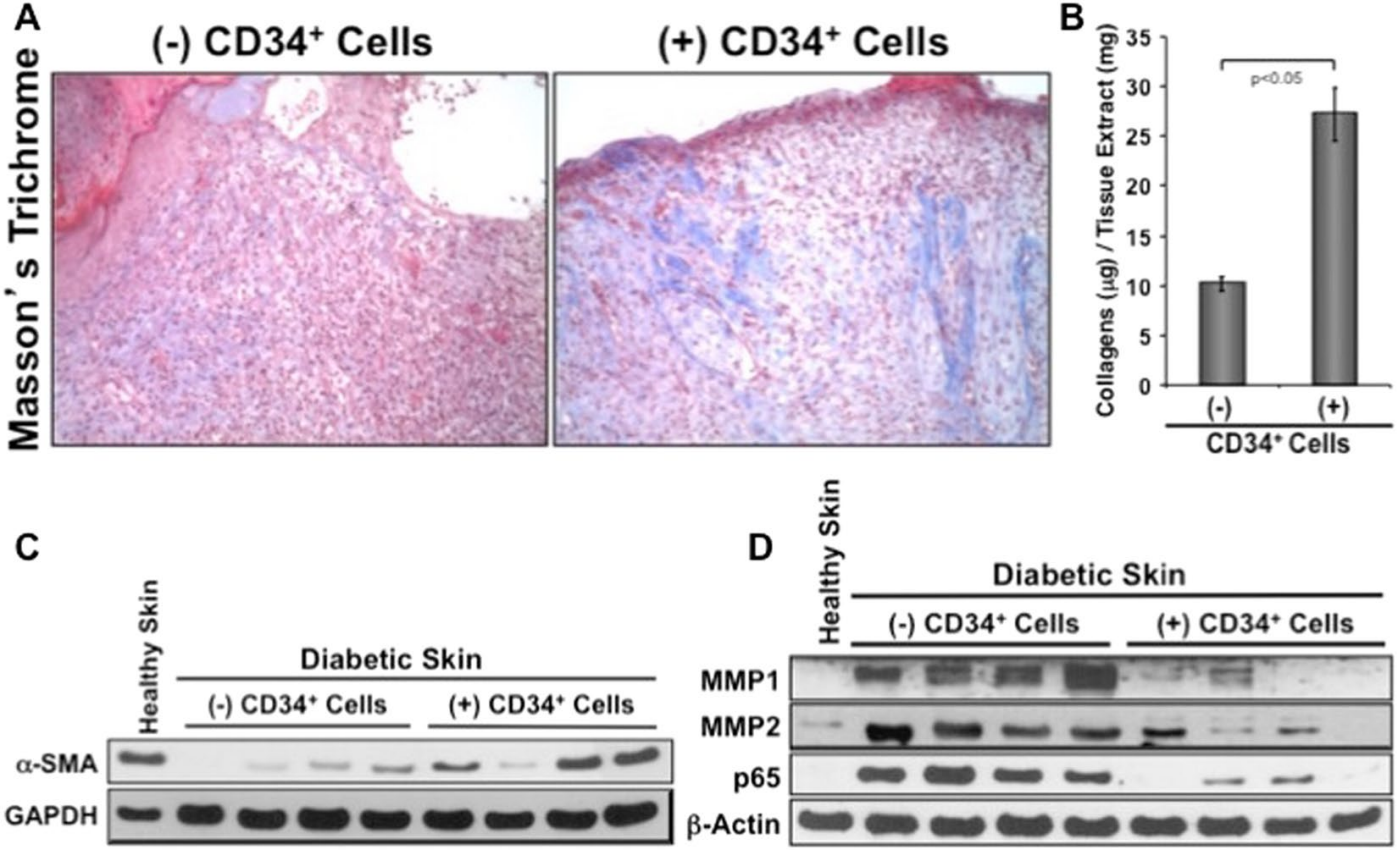
Increased collagen deposition, fibroblast recruitment, and decreased MMPs in wounds after CD34^+^ cell therapy. (**A**) Blue stain represents collagen with Masson’s Trichrome staining after CD34^+^ cells therapy at day10 (right panel), and control on the left. (**B**) Graphical presentation of the total collagen content in wound tissue lysates at day 10 with or without cell therapy. Data are presented as mean ± SEM (n = 3). (**C**) Representative western blot analysis of alpha smooth muscle actin (α-SMA) expression in skin tissue extracts at day 5 with or without cell therapy (n = 4). GAPDH expression was used as a loading control. (**D**) Representative western blot images for MMP-1, -2, and NF-κB (p65) protein levels in wound tissue lysates at day 5 with or without cell therapy (n = 4). β-actin was used as loading control.

To explore the function of CD34^+^ cell therapy in extracellular matrix formation, we performed histochemical analysis of the wound sections by using Masson’s trichrome staining. Staining showed that use of CD34^+^ cells increased deposition of collagen (blue staining) in wound tissues of diabetic mice compared with control at day 10 (Fig. 6A) indicating restoration of normal ECM formation. In addition, quantitation of total collagen showed that significantly (p < 0.05) higher (27.37 μg/mg of total collagen in treatment group, vs. 10.30 μg/mg in control group) amount of collagen in diabetic mice treated with CD34^+^ cells than that of control (Fig. 6B). These data indicate that CD34^+^ cell therapy resulted in increased amount of collagen at the wound bed in diabetic mice, facilitating wound closure.

### Effect of CD34^+^ cell therapy in recruiting fibroblasts in the wound

To explore the effect of CD34^+^ cell therapy in attracting fibroblasts at the wound area, western blot analysis was performed with wound tissues proteins by using α-SMA, a marker for fibroblast cells. The level of the α-SMA molecule was higher in wound tissues from mice infused with CD34^+^ cells compared to media-treated control mice (Fig. 6C), indicating that CD34^+^ cell therapy enhanced fibroblast migration to the effected wound that further helped in wound contraction.

### Effect of CD34^+^ cell therapy on wound tissue matrix metalloproteinases and NF-κB

We next sought to assess the expression levels of collagenase (MMP-1) and gelatinase (MMP-2) in the wound tissues of animals receiving CD34^+^ cells or vehicle only using western blot methods. There is a remarkable reduction in the levels of MMP-1 and MMP-2 in wound tissues of the animals received CD34^+^ cells at day 5 compared to the wounds of animals received no cells (Fig. 6D). Thus, reduced levels of MMP-1 and MMP-2 at the wounds after CD34^+^ cell injection might make an important contribution to the collagen deposition, which helped in accelerated wound closure. In addition, a remarkable reduction in the level of NF-κB in samples of the animals received CD34^+^ cells at day 5 were noticed compared with wounds of control animals (Fig. 6D). Thus, reduced levels of NF-κB at the wound after infusion of CD34^+^ cells might have a critical regulatory role in down regulating inflammatory and MMP molecules at the wound bed, which in turn contributed to faster wound closure.

### Effect of CD34^+^ cells on TNF-α-stimulated MMP-1 expression in dermal fibroblasts

**Figure 7:**
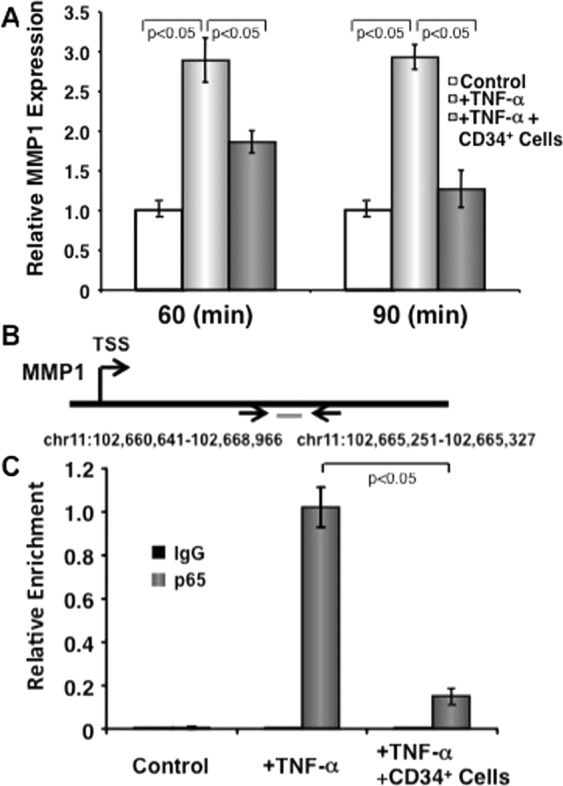
CD34^+^ cells inhibit TNF-α-induced NF-κB-mediated MMP-1 transcription in dermal fibroblasts. (**A**) CD34^+^ cells inhibit TNF-α-induced MMP1 expression in dermal fibroblasts. Human primary dermal fibroblast cells were cultured either in media alone or in presence or absence of CD34^**+**^ cells (1:1 ratio and CD34^**+**^ cells were removed after culture) plus in presence or absence of TNF-α (10 ng/ml) for 60 or 90 minutes. Quantitative RT-PCR was performed in fibroblasts for MMP-1. Values were normalized to GAPDH levels. Error bars represent mean ± SEM (representative of 3 independent experiments in triplicate). (**B**) Schematic illustration of the MMP-1 gene for transcriptional start site (TSS), and the location of the probe designed for ChIP analysis. (**C**) Human primary dermal fibroblast cells were cultured in presence or absence of CD34^**+**^ cells (1:1 ratio and CD34^**+**^ cells were removed after culture) and presence or absence of TNF-α (10 ng/ml) for 60 minutes for ChIP assays. Fibroblasts treated with TNF-α or TNF-α plus CD34^**+**^ cells or fibroblasts alone for 60 min were immunoprecipitated with NF-κB (p65) antibody. Immunoprecipitated chromatin from three independent experiments was analyzed by quantitative PCR for the presence of MMP-1 genomic DNA that was normalized to the respective input levels. Error bars represent Mean ± S.D.

As higher abundance of TNF-α and MMP-1 was observed is diabetic wound tissues, we wanted to confirm if CD34^+^ cells have any effect on MMP-1 in human dermal fibroblast under the influence of TNF-α. Quantitative RT-PCR analysis demonstrated that MMP-1 expression was almost 3-fold higher when treated with TNF-α for 60 minutes. However, in the presence of CD34^+^ cells, this induction of MMP-1 was significantly abrogated (Fig. 7, upper panel). Similar results were also observed at the 90-minute time point. This data confirms that the CD34^+^ cells have ability to regulate MMP-1 in inflammatory conditions.

### Effect of CD34^+^ cells on NF-κB-mediated MMP-1 transcription in dermal fibroblasts

As NF-κB-like elements were identified in the MMP-1 promoter region^20^ and NF-κB activity is indispensable for MMP-1 upregulation^21^, we wanted to confirm whether CD34^+^ cell mediated suppression of MMP-1 transcription in response to TNF-α is NF-κB mediated using ChIP analysis. A probable NF-κB binding site with in the MMP-1 locus was identified using publicly available datasets (Fig. 7, middle panel)^22^. Three independent ChIP experiments demonstrated that the recruitment of NF-κB to this region was dramatically increased in TNF-α treated dermal fibroblasts. However, the presence of CD34^+^ cells inhibited recruitment of NF-κB in the MMP-1 locus (Fig. 7, lower panel). This result confirms that CD34^+^ stem cells indeed regulate MMP-1 promoter region of NF-κB binding sites to reduce inflammation.

## Discussion

Although many advanced therapeutic options are available for management of diabetic wounds, a significant number of patients undergo amputation due to the failure of therapy, suggesting a dire need for more effective alternative therapeutic developments^23^. In the past several years, various attempts have been made to treat diabetic wounds with autologous stem cells with limited success^24^. Autologous stem cell therapy may not be a suitable option for treating wounds, especially in the aged population, because advanced age negatively impacts stem cell survival and function^25,26^. Cord blood derived adult stem cells are advantageous in treating various degenerative disorders because of their multipotent nature and reduced immunogenicity. However, the amount of stem cells procured from a single cord blood unit is insufficient for any clinical or preclinical applications. We were successful in expanding human umbilical cord blood-derived CD34^+^ cells using aminated PES nanofiber coated plates while preserving their stemness and their biological functionality^16,27^. Aminated PES nanofiber coated plates have several advantages over polystyrene culture plates by providing three dimensional (3D) configurations mimicking bone marrow micro structures, which supports expansion of hematopoietic stem cells preserving their stemness^28^. Whereas, culturing hematopoietic stem cells on polystyrene plates do not support large expansion, and do not preserve stemness of the expanded cells. The potential of these aminated PES nanofiber expanded CD34^+^ stem cells in treating diabetic wounds and the underlying mechanism is yet to be defined.

Chemical induction of diabetes is a well-established method, and not only provides a simple, reliable and cheap model of diabetes in rodents^29^, but is also useful for transplantation studies^18^. We have developed a stable diabetic condition in immunocompromised NOD/SCID mice using STZ injection where human cells could be transplanted and efficiently integrated without immune rejection. Induced cutaneous wounds took longer to heal in these diabetic mice than normal mice^13,14^, most likely due to impaired angiogenesis, re-epithelialization and cellularity at the effected wound. However, CD34^+^ stem cell therapy resulted in improved wound closure in diabetic mice compared to vehicle treated controls (Fig. 2). As homing (CXCR4) and adhesion (LFA-1) markers expressed highly on the nanofiber-expanded CD34^+^ cells^16^, we next explored whether in diabetic mice systemically transplanted CD34^+^ stem cells migrated to the wound bed. These cells were indeed migrated to the wound area at the early time points and gradually decreased in number as healing progressed (Fig. 3A), and mediated greater cellularization within the wounded tissues (Fig. 3B). It is established that diabetes impairs inherent HSC mobilization by altering niche function^30^. Considering the inherent disability of HSC function in diabetic condition, allogeneic CD34^+^ cell transplantation contributed significantly to accelerated wound closure in diabetic NOD/SCID mice.

Although inflammation is necessary in the early stage of the healing process, persistent and unresolved inflammation is a major cause of failure in diabetic wound healing, and neutrophils are among the key inflammatory regulators. Infiltrated neutrophils stay at the wound bed and secrete proinflammatory cytokines such as IL-1β and TNF-α, which further activates downstream molecular cascades^31,32^. We observed a higher neutrophil abundance in the wound bed at the later stage of the wound healing process (day 10) in diabetic NOD/SCID mice, while a significantly reduced number of neutrophils were found after CD34^+^ cell therapy (Fig. 4B,C). Proinflammatory activity at the diabetic wound bed was further supported by enhanced levels of IL-1β and TNF-α in the wound tissues of the control diabetic animals compared to the CD34^+^ treated animals, which correlates nicely with the improved wound healing after treatment of diabetic mice with CD34^+^ cells. On the other hand, IL-10 is among the prominent anti-inflammatory cytokines released following inflammation that helps in wound resolution and healing^33^. Here, CD34^+^ cell treatment-mediated resolution of wound inflammation in diabetic NOD/SCID mice can be partially explained by the presence of significantly higher level of IL-10 in the injured wound (Fig. 4A). Persistent inflammation also reduces angiogenesis^7^. We further assessed whether angiogenesis can be improved by CD34^+^ stem cell therapy in hyperglycemic mice. Wound angiogenesis was indeed enhanced significant after CD34^+^ cell therapy in hyperglycemic mice (Fig. 5A,B).

After wounding, fibroblasts that are residing in the wound edge, proliferate and move into the provisional matrix that is made with the clots of fibrin^34^. The connective tissue replaces provisional matrix called granulation tissue, which is made of in combination with small blood vessels, extracellular matrix, and fibroblast cells from where α-smooth muscle actin (α-SMA) expressing myofibroblasts can be differentiated^34,35^. Myofibroblasts heal the wound tissue through their strong contractile force^36^. Wound contraction is impaired in diabetic patients because of impaired dermal fibroblast proliferation and reduced α-SMA expression in these fibroblasts. Elevated levels of TNF-α present in diabetic wounds might contribute to reduce healing efficacy, as TNF-α suppresses α-SMA in human dermal fibroblasts^11^. We found that application of CD34^+^ cells enhanced expressions of α-SMA in the injured wound of diabetic mice (Fig. 6C). The increased expressions of α-SMA is most likely due to increased number of smooth muscle cells at the wound area after CD34^+^ cells therapy that helped maintain normal wound contraction and facilitated wound closure in diabetic mice. Dermal fibroblasts also secret collagen proteins, which are major components of skin ECM^37^. Hyperglycemia hinders the secretion of ECM components from fibroblasts^8,9^. Here we show that total collagen content significantly increased after CD34^+^ cell treatment (Fig. 6A,B). This increased amount of collagen at the wounded skin may occur due to decreased MMP activity at the effected wound after CD34^+^ cell therapy in diabetic mice^12^. We have found that expression of MMPs, especially MMP-1 and MMP-2, is high in the diabetic wound bed. However, application of CD34^+^ cells reduced amount of MMP-1 and MMP-2 at the diabetic wound tissues (Fig. 6D). Collectively, present data indicate that CD34^+^ cell therapy reduced the levels of MMPs, restored fibroblast migration in the diabetic wounds that in turn contributed to effective ECM deposition. Increased ECM formation by CD34^+^ cell therapy might have achieved by resolving inflammation through decreased neutrophil abundance and diminished quantities of TNF-α and IL-1β, and boosted IL-10 expression.

MMP1 is one of the predominant collagenase found in chronic wounds, which has collagenolytic activity^12^. Excess of TNF-α and IL-1β in unresolved inflammatory environment trigger signals for MMP expression via NF-κB pathway^21,38^. To define molecular mechanisms by which MMPs are being regulated, we found a decreased abundance of NF-κB along with TNF-α and IL-1β in diabetic wounds after CD34^+^ cell therapy, indicating a CD34^+^ cell-mediated regulatory interaction between NF-κB and MMP-1 expression. We confirmed this interaction by *in vitro* CD34^+^ cells co-culture with dermal fibroblasts and showed that amount of MMP-1 in dermal fibroblasts is downregulated in addition of TNF-α stimulation (Fig. 7, upper panel). Further ChIP analysis confirmed CD34^+^ cell-mediated suppression of NF-κB regulated MMP-1 transcription in dermal fibroblasts after addition of TNF-α stimulation (Fig. 7, lower panel). These findings are correlated with the previous studies where it has been observed that NF-κB activity is necessary for MMP-1 increment in dermal fibroblasts of rabbit^21^. Thus, CD34^+^ cells were able to downregulate MMP-1 expression by targeting the NF-κB-mediated transcriptional activity. Thus, our confirmatory *in vitro* data support our previous *in vivo* finding that CD34^+^ stem cells may regulate the expression of MMPs by suppressing key master transcriptional factor NF-κB and further transcription of its downstream genes in the inflammatory milieu in diabetic condition.

## Conclusion

In conclusion, we have demonstrated the efficacy of CD34^+^ cell therapy for healing of cutaneous wounds in mice with diabetes, which occurred by resolving inflammation, increasing, angiogenesis, enhancing epithelialization and improving granulation tissue formation. Mechanistically, these cells modulate catabolic activity of matrix metalloproteinases by regulating the NF-κB signaling pathway. Therefore, umbilical cord blood-derived CD34^+^ cells expanded on nanofiber scaffold might be considered a promising stem cell source for future cell-based therapy for diabetic wounds.

## Supplementary information

Supplementary information
