# Supplementary material for: Nanofiber-expanded human CD34+ cells heal cutaneous wounds in streptozotocin-induced diabetic mice
Source: Sci Rep. 2019 Jun 10;9:8415. doi: 10.1038/s41598-019-44932-7 (PMC6557810; doi:10.1038/s41598-019-44932-7)
Supplement: Supplementary file 1 — Supplementary information [file 41598_2019_44932_MOESM1_ESM.pdf]

Nanofiber-expanded human CD34+ cells heal cutaneous wounds in streptozotocin-induced diabetic mice

Suman Kanji[1, 4], Manjusri Das[1], Matthew Joseph[1], Reeva Aggarwal[1], Sudarshana M. Sharma[2], Michael Ostrowski[2], Vincent J. Pompili[1], Hai-Quan Mao[3], and Hiranmoy Das[1, 4*]

Supplementary Figure 1.

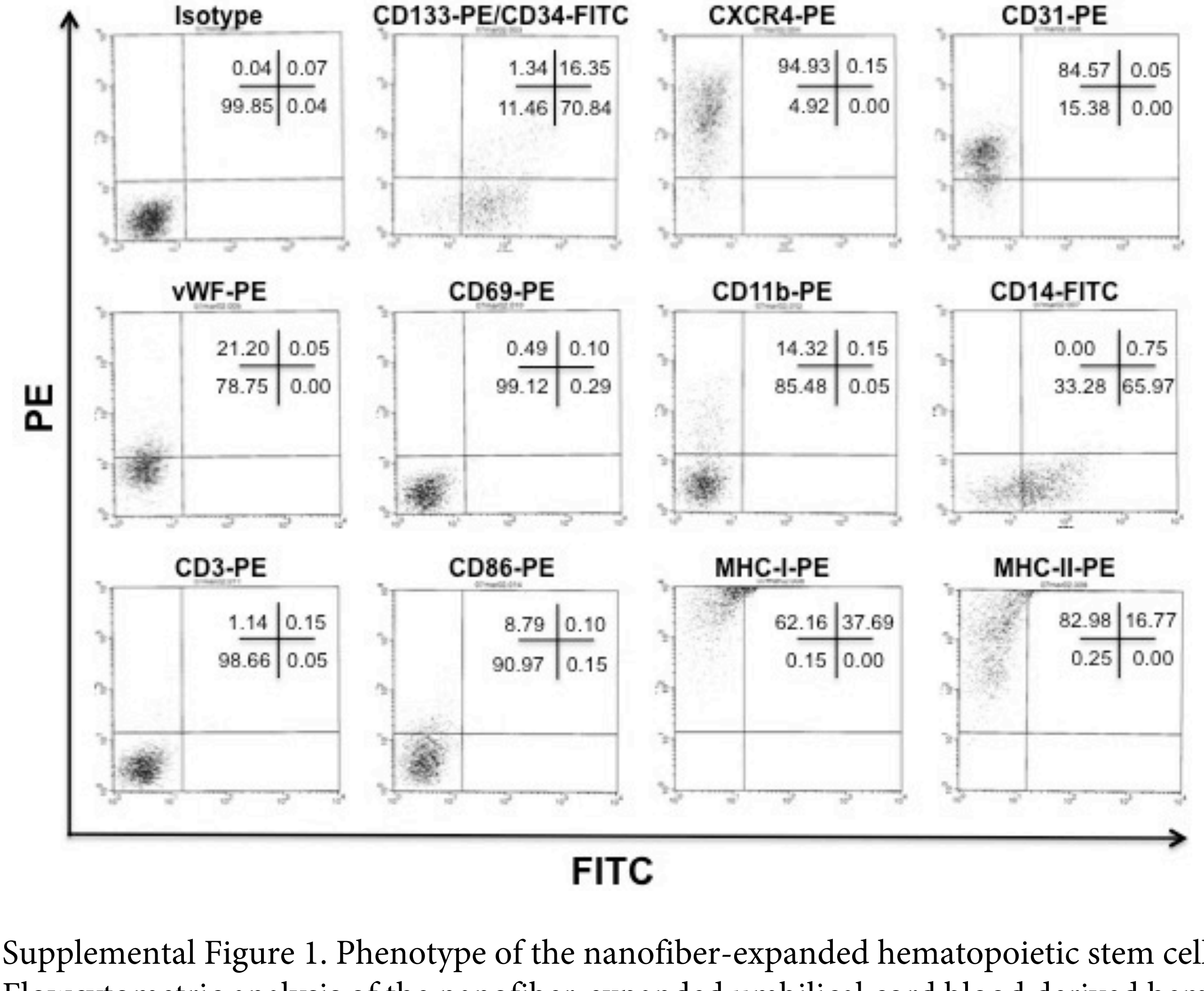

Supplemental Figure 1. Phenotype of the nanofiber-expanded hematopoietic stem cells. Flowcytometric analysis of the nanofiber-expanded umbilical cord blood derived hematopoietic stem cells (HSC). Following are the markers description: CD133 = primitive HSC marker; CD34 = mature HSC marker; CD31 and vWF = early endothelial cell markers; CD69 = HSC activation marker; CD11b, and CD14 = myeloid cell markers; CD3 = Pan T cell marker, CD86 = macrophage marker, MHC-I and MHC-II = antigen presentation molecules.

Nanofiber-expanded human CD34+ cells heal streptozotocin-induced murine diabetic wounds by epigenetically regulating MMP1

Suman Kanji[1], 4, Manjusri Das[1], Matthew Joseph[1], Reeva Aggarwal[1], Sudarshana M. Sharma[2], Michael Ostrowski[2], Vincent J. Pompili[1], Hai-Quan Mao[3], and Hiranmoy Das[1, 4*]

Western blot scanned data

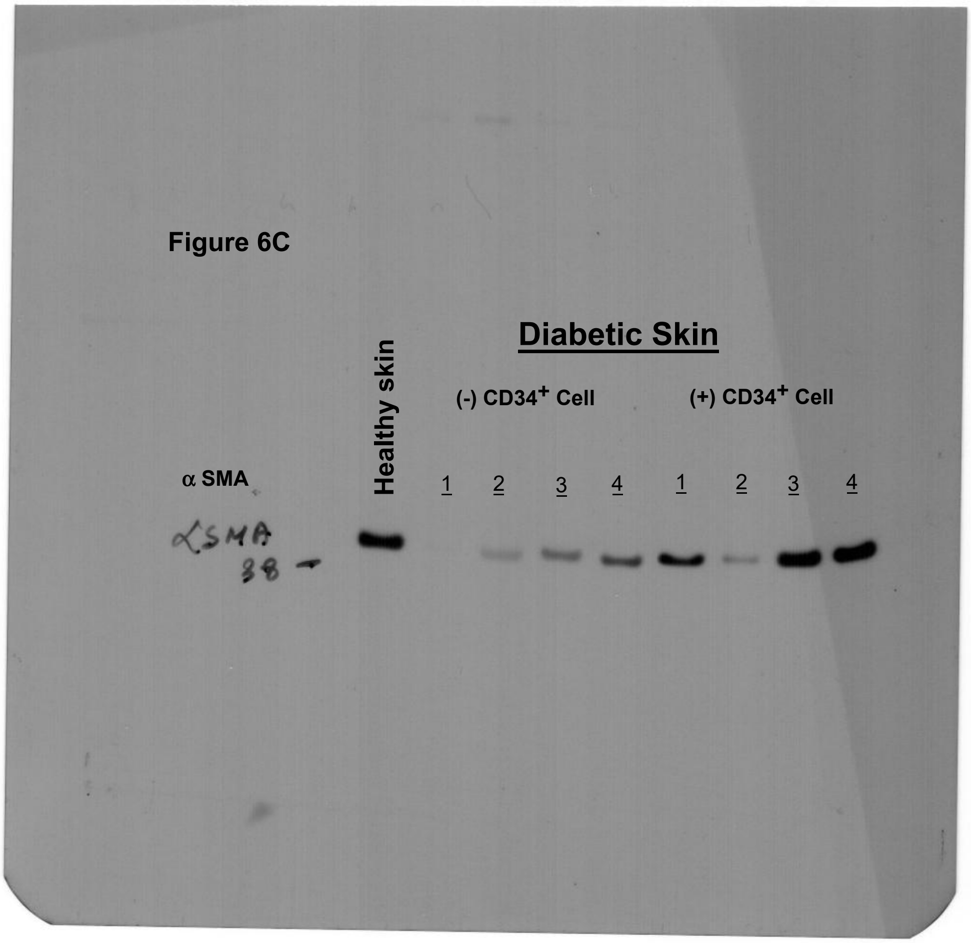

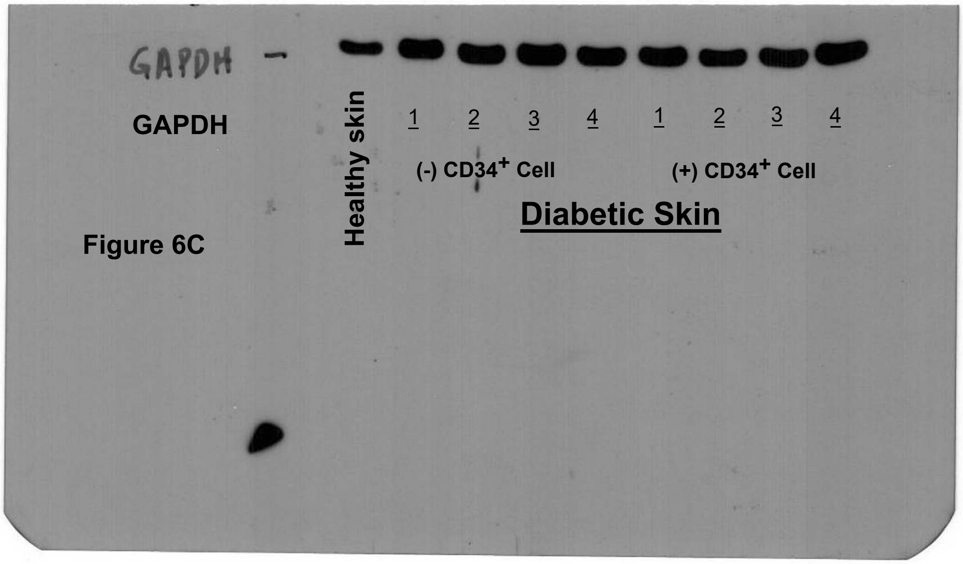
GAPDH
GAPDH
Healthy skin
1 2 3 4 1 2 3 4
(-) CD34+ Cell
(+) CD34+ Cell
Diabetic Skin
Figure 6C

Figure 6D

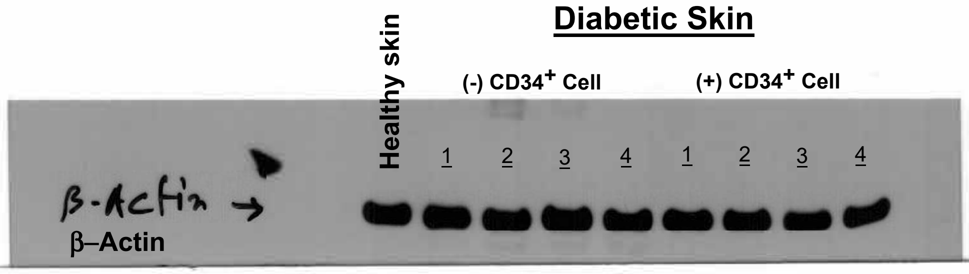
Diabetic Skin
Healthy skin
(-) CD34+ Cell
(+) CD34+ Cell
1
2
3
4
1
2
3
4
β-Actin →
β–Actin

Figure 6D

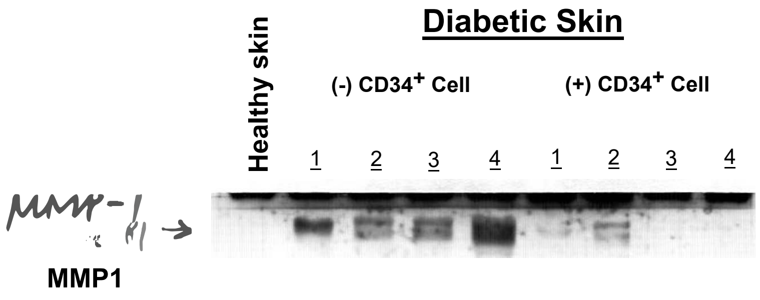

Healthy skin
Diabetic Skin
(-) CD34+ Cell
(+) CD34+ Cell
1 2 3 4 1 2 3 4
MMP1

Figure 6D

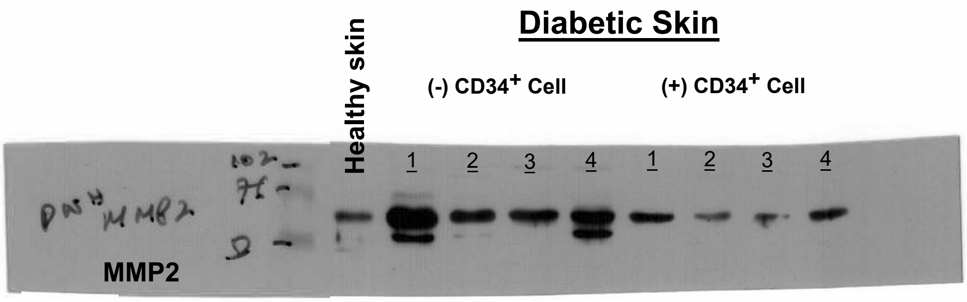
Diabetic Skin
Healthy skin
(-) CD34+ Cell
(+) CD34+ Cell
1
2
3
4
1
2
3
4
MMP2

Figure 6D

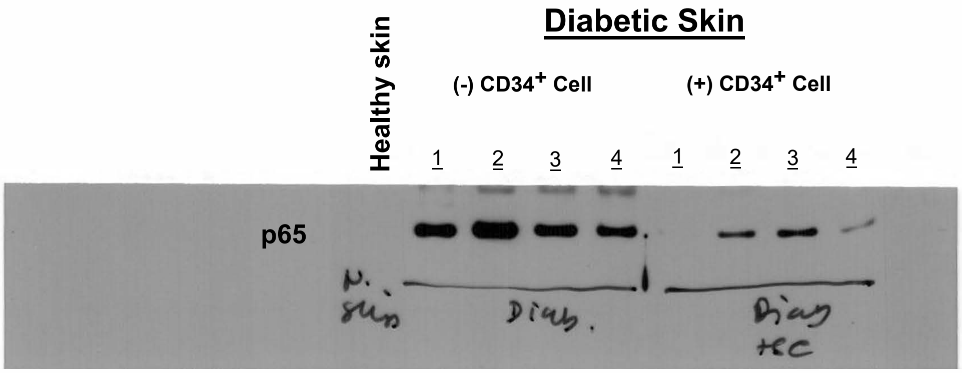
Diabetic Skin
Healthy skin
(-) CD34+ Cell
(+) CD34+ Cell
1
2
3
4
1
2
3
4
p65
